# The Definition of Insulin Resistance Using HOMA-IR for Americans of Mexican Descent Using Machine Learning

**DOI:** 10.1371/journal.pone.0021041

**Published:** 2011-06-14

**Authors:** Hui-Qi Qu, Quan Li, Anne R. Rentfro, Susan P. Fisher-Hoch, Joseph B. McCormick

**Affiliations:** 1 Division of Epidemiology, Human Genetics and Environmental Sciences, Brownsville Regional Campus, The University of Texas School of Public Health, Brownsville, Texas, United States of America; 2 Endocrine Genetics Lab, The McGill University Health Center, Montreal Children's Hospital, Montréal, Québec, Canada; 3 College of Nursing, University of Texas at Brownsville and Texas Southmost College, Brownsville, Texas, United States of America; Mayo Clinic, United States of America

## Abstract

**Objective:**

The lack of standardized reference range for the homeostasis model assessment-estimated insulin resistance (HOMA-IR) index has limited its clinical application. This study defines the reference range of HOMA-IR index in an adult Hispanic population based with machine learning methods.

**Methods:**

This study investigated a Hispanic population of 1854 adults, randomly selected on the basis of 2000 Census tract data in the city of Brownsville, Cameron County. Machine learning methods, support vector machine (SVM) and Bayesian Logistic Regression (BLR), were used to automatically identify measureable variables using standardized values that correlate with HOMA-IR; K-means clustering was then used to classify the individuals by insulin resistance.

**Results:**

Our study showed that the best cutoff of HOMA-IR for identifying those with insulin resistance is 3.80. There are 39.1% individuals in this Hispanic population with HOMA-IR>3.80.

**Conclusions:**

Our results are dramatically different using the popular clinical cutoff of 2.60. The high sensitivity and specificity of HOMA-IR>3.80 for insulin resistance provide a critical fundamental for our further efforts to improve the public health of this Hispanic population.

## Introduction

The homeostasis model assessment-estimated insulin resistance (HOMA-IR), developed by Matthews et al. [1] has been widely used for the estimation of insulin resistance in research. Compared with the “gold” standard euglycemic clamp method for quantifying insulin resistance [2], quantification using HOMA-IR is more convenient. It is calculated multiplying fasting plasma insulin (FPI) by fasting plasma glucose (FPG), then dividing by the constant 22.5, i.e. HOMA-IR = (FPI×FPG)/22.5 [3]. This method has been applied across all ethnic groups. One study suggested that the range of normal HOMA-IR in a healthy Hispanic population may be higher than for Caucasians in central and north America [3], and certainly this population is known to have a genetic susceptibility to type 2 diabetes, which is closely associated with insulin resistance. Therefore, in spite of its importance, the lack of standardized reference range for HOMA-IR has hindered its clinical and population application. In order to address this issue, we developed a computational approach to define the reference range of HOMA-IR in Mexican Americans by identifying factors that are associated with HOMA-IR. We used the accepted national standard values of the variables in our model (e.g. BMI, waist-to-hip ratio, triglyceride levels etc) based on published recommendations that are currently and widely used in different populations. Using this method we identified those variables associated with elevated HOMA-IR and then defined its optimal reference range in an adult Hispanic (Mexican American) population in south Texas.

## Methods

### Ethics Statement

Written informed consent was obtained from each participant, and the Committee for the Protection of Human Subjects of the University of Texas Health Science Center at Houston (UTHealth) approved this study.

### Subjects

This study used data from 1854 adult individuals with HOMA-IR values from the Cameron Cohort Hispanic Cohort (CCHC). These individuals over 18 years of age were randomly selected for recruitment to the study on the basis of 2000 Census tract data in the city of Brownsville, Cameron County, over 90% of whom are Mexican American. The design and collection of data for this cohort was previously described [4].

### Identification of HOMA-IR correlated factors

Two machine learning methods, the support vector machine (SVM, http://www.csie.ntu.edu.tw/~cjlin/libsvm) [5] and Bayesian Logistic Regression (BLR, http://code.google.com/p/bbrbmr/), were used to automatically capture HOMA-IR correlated factors. The following variables were included in our risk model (methods described in our previous report [4]): gender, age, body mass index (BMI), waist/hip ratio, FPG, blood pressure, physical activity, alcohol consumption, smoking, education levels, self- reported history of hepatitis, fasting serum lipids [serum triglycerides, total cholesterol, high-density lipoprotein (HDL) cholesterol, and low-density lipoprotein (LDL) cholesterol], and serum transaminases [alanine aminotransferase (ALT) and aspartate aminotransferase (AST) all conducted in a CLIA approved laboratory]. Insulin was measured in serum frozen at −80°C within 1 hour of taking the sample. Insulin was measured in batches using the enzyme-linked immunosorbent assay insulin kit (Mercodia, Uppsala, Sweden) using the standard curves supplied with the kit [4].

### Statistical analysis

Using the HOMA-IR correlated factors identified by SVM and BLR, the 1854 individuals were clustered by the K-means method (IBM SPSS 19.0 software). The significance of each attribute between the two K-means clusters was tested by ANOVA. Based on the classification results, a series of cutoffs of HOMA-IR was evaluated for the sensitivity (the true positive rate) and specificity (the true negative rate, or 1- the false positive rate). To identify the best cutoff value, a receiver operator characteristic (ROC) analysis was performed based on the sensitivity and specificity values of the series of cutoffs. The best cutoff was identified using the maximum Matthews correlation coefficient.

## Results and Discussion

Using the supervised machine learning methods SVM and BLR, we identified five groups of factors correlated with increased HOMA-IR (Table S1 and Figure S1), including, BMI and waist-hip ratio, FPG, plasma lipids, hypertension and liver enzymes. BMI had the largest effect correlated with HOMA-IR. Waist/hip ratio contributes an additional independent effect, which emphasizes the important role for central fat distribution in the risk of insulin resistance. Increased FPG is a direct result of insulin resistance because of decreased sensitivity to the glucose-lowering effect of insulin. Both serum triglycerides and total cholesterol were associated with HOMA-IR, though the effect of triglycerides is stronger than cholesterol. Both elevated diastolic blood pressure and elevated systolic blood pressure were associated with HOMA-IR. ALT is mainly produced in the liver, and is elevated in serum in conditions leading to chronic hepatocellular injury. Elevated AST may also reflect liver injury, but less specifically. The correlation between liver function and insulin resistance may be explained by the critical role of liver in glucose-insulin metabolism [6], liver injury caused by insulin resistance [7], or disorders of adipose metabolism compounded by liver dysfunction and insulin resistance [8], [9]. The identification of these factors closely correlated with HOMA-IR in the SVM model enables us to remove the factors that are not associated with increased HOMA-IR. Otherwise, the noise effects of uncorrelated factors interfere with the proper classification of individuals with or without insulin resistance. There was no significant association of gender or age with HOMA-IR in the model such that we did not include either among the variables that best define HOMA-IR. Thus we are able to use the HOMA-IR reference range for the entire adult population.

**Figure 1:**
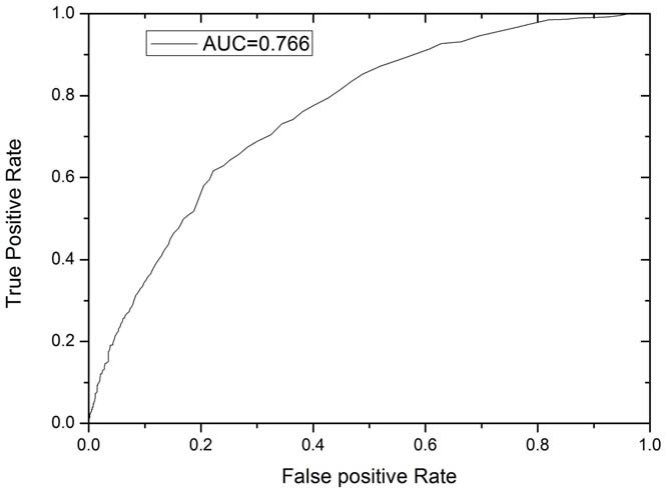
The ROC curve for the identification of the best cutoff value of HOMA-IR. X-axis represents false positive (FP) rate (or 1-specificity); Y-axis represents true positive (TP) rate (sensitivity).

After the identification of factors which best correlated with elevated HOMA-IR we used the reference ranges of these factors to classify the individuals as having insulin resistance (Table S2). The reference ranges of BMI, serum triglycerides, total cholesterol, HDL cholesterol, and blood pressures, were based on the recommendations of the American Heart Association (www.americanheart.org). The reference range of FPG was based on the 2010 Clinical Practice Recommendations of the American Diabetes Association (ADA) [10]. Based on these categorized factors, the 1854 individuals were classified into two groups by the K-means clustering (Table 1). Group 1 is comprised of 795 individuals, corresponding to those with insulin resistance; Group 2 is comprised of 1059 individuals, corresponding to the control group. Based on the K-means classification results, the sensitivity and specificity of a series of HOMA-IR cutoff values was tested (Table 2 and Fig. 1). The fine-scans of the series of HOMA-IR cutoff values are shown in detail in Table S3. Using these data we determined that the most relevant cutoff for Mexican Americans was a HOMA-IR = 3.80. This cut-off had a specificity = 0.778 and sensitivity = 0.616 for identifying insulin resistance. Among the factors used for the K-means classification, HDL cholesterol was the only protective factor, which had also the least statistical significance (Table 1). However, the inclusion of HDL cholesterol increased the correlation between HOMA-IR and the K-means clusters obviously, i.e. AUC = 0.766 with HDL cholesterol, and AUC = 0.721 without HDL cholesterol, while both conditions had the same optimal cutoff of HOMA-IR = 3.80.

**Table 1:** K-means classification of the 1854 individuals^a^.

Attribute	Group 1 center (n = 795)	Group 2 center (n = 1059)	ANOVA test *P* value
BMI	2.85	2.08	5.23×10^−85^
waist/hip ratio	1.95	1.81	4.21×10^−18^
FPG	1.90	1.29	3.01×10^−74^
Serum triglycerides	2.47	1.12	<10^−100^
Total cholesterol	1.72	1.28	2.28×10^−43^
HDL cholesterol	1.54	1.46	1.32×10^−3^
Systolic blood pressure	1.82	1.27	1.29×10^−57^
Diastolic blood pressure	1.42	1.12	1.45×10^−33^
ALT	1.45	1.29	2.75×10^−13^
AST	1.33	1.25	5.74×10^−4^

a Each attribute was graded based on the recommendations of the American Heart Association (www.americanheart.org) or the American Diabetes Association (ADA) [10]. Details are shown in Table S2.

BMI: body mass index; FPG: fasting plasma glucose; HDL: high-density lipoprotein; ALT: alanine aminotransferase; AST: aspartate aminotransferase.

**Table 2:** The specificity and sensitivity for insulin resistance syndrome of various HOMA-IR cutoff values.

HOMA-IR cutoff	Sensitivity	Specificity	MCC
0.30	1.000	0.004	0.040
0.55	0.999	0.021	0.087
0.80	0.996	0.054	0.140
1.05	0.991	0.112	0.204
1.30	0.985	0.180	0.262
1.55	0.960	0.261	0.293
1.80	0.932	0.336	0.320
2.05	0.906	0.408	0.348
2.30	0.872	0.480	0.370
2.55	0.825	0.544	0.375
2.80	0.775	0.600	0.373
3.05	0.737	0.641	0.375
3.30	0.688	0.700	0.385
3.55	0.653	0.740	0.393
3.80	0.616	0.778	0.400
4.05	0.560	0.800	0.372
4.30	0.517	0.813	0.348
4.55	0.494	0.836	0.354
4.80	0.463	0.849	0.342
5.05	0.428	0.863	0.327
5.30	0.395	0.878	0.317
5.55	0.366	0.889	0.304
5.80	0.351	0.898	0.303
6.05	0.333	0.907	0.299
6.30	0.318	0.913	0.294
6.55	0.294	0.920	0.281
6.80	0.279	0.927	0.278
7.05	0.263	0.935	0.274
7.30	0.257	0.939	0.274
7.55	0.240	0.943	0.266
7.80	0.225	0.947	0.256

Because of the extremely high correlation between HOMA-IR and FPI (r^2^ = 0.798), FPI levels were not used in the above procedures. However, to confirm our results, we tested the effect of introducing FPI into the K-means classification. The introduction of the FPI attribute dramatically increased the performance of the machine learning methods, i.e. AUC = 0.986 for SVM, and AUC = 0.910 for BLR. A widely used cutoff of FPI≥12 mU/L as abnormal was adopted for the categorization of FPI [11], [12]. The K-means clustering classified 844 individuals as having insulin resistance, and 1010 individuals as normal controls. The fine-scans of the series of HOMA-IR cutoff values (AUC = 0.809) showed exactly the same best cutoff of HOMA-IR = 3.80 with specificity = 0.818 and sensitivity = 0.641.

In summary, our study showed the best cutoff of HOMA-IR in Mexican Americans to be 3.80 for the definition of insulin resistance. This is higher than the widely adopted cutoff of 2.60 [12] for which we calculate a specificity of only 0.552 and sensitivity of 0.814. Our model suggests that the lower cut-off will misclassify 44.8% as having insulin resistance syndrome. To compromise we suggest the reference values for HOMA-IR in Mexican Americans as HOMA-IR<2.60 as the normal range, HOMA-IR 2.60–3.80 as “borderline high” without labeling these individuals as having insulin resistance, and HOMA-IR>3.80 as “high” having clear correlates of insulin resistance. Using this standard, 39.5% of the adult Cameron County Hispanic population have HOMA-IR<2.60; that is, normal. 21.4% have HOMA-IR 2.60–3.80; that is, borderline. 39.1% have HOMA-IR>3.80; that suggests insulin resistance. In doing this we now differentiate 21.4% of the population as having borderline high HOMA-IR from the 39.1% population with more obvious insulin resistance, thus dramatically increasing the specificity and usefulness of HOMA-IR for targeting research and intervention. This distinction will be useful in studies of this population known to have high genetic predisposition for diabetes [13], and in whom the range of HOMA-IR values is likely to be higher than other populations with lower genetic susceptibility. It is worth noting that the computational approach of this study reminds us to be cautious in applying this reference in other populations. The reference defined by our study may help to clear the confusion on the clinical application of HOMA-IR in Mexican Americans, and will refine clinical decisions on appropriate diagnosis or treatment of the insulin resistance syndrome. Since the insulin resistance syndrome is a major public health issue in this population living poor socio-economic conditions, we may use it in the design of clinical trials preventing progression from borderline to high HOMA-IR. This reference will be fundamental to our further efforts to improve population health with optimal cost-benefit ratios.

## Supporting Information

Figure S1 The performances of machine learning methods in the identification of HOMA-IR corrected factors in the Cameron Cohort Hispanic Cohort (CCHC). (a) The SVM model; (b) The BLR model. As shown by the area under the receiver operator characteristic curve (AUROC) scores, both methods have good performance in modeling the HOMA-IR corrected factors, while the SVM model (AUC = 0.816) has slightly better performance than the BLR model (AUC = 0.800).
(DOCX)

Table S1 Identification of HOMA-IR corrected factors by SVM and BLR.
(DOCX)

Table S2 Reference ranges of the HOMA-IR corrected factors.
(DOCX)

Table S3 The specificity and sensitivity for insulin resistance syndrome of fine-scans of the series of HOMA-IR cutoff values.
(DOCX)
