# Supplementary figures and images for: The Definition of Insulin Resistance Using HOMA-IR for Americans of Mexican Descent Using Machine Learning
Source: PLoS One. 2011 Jun 14;6(6):e21041. doi: 10.1371/journal.pone.0021041 (PMC3114864; doi:10.1371/journal.pone.0021041)

**
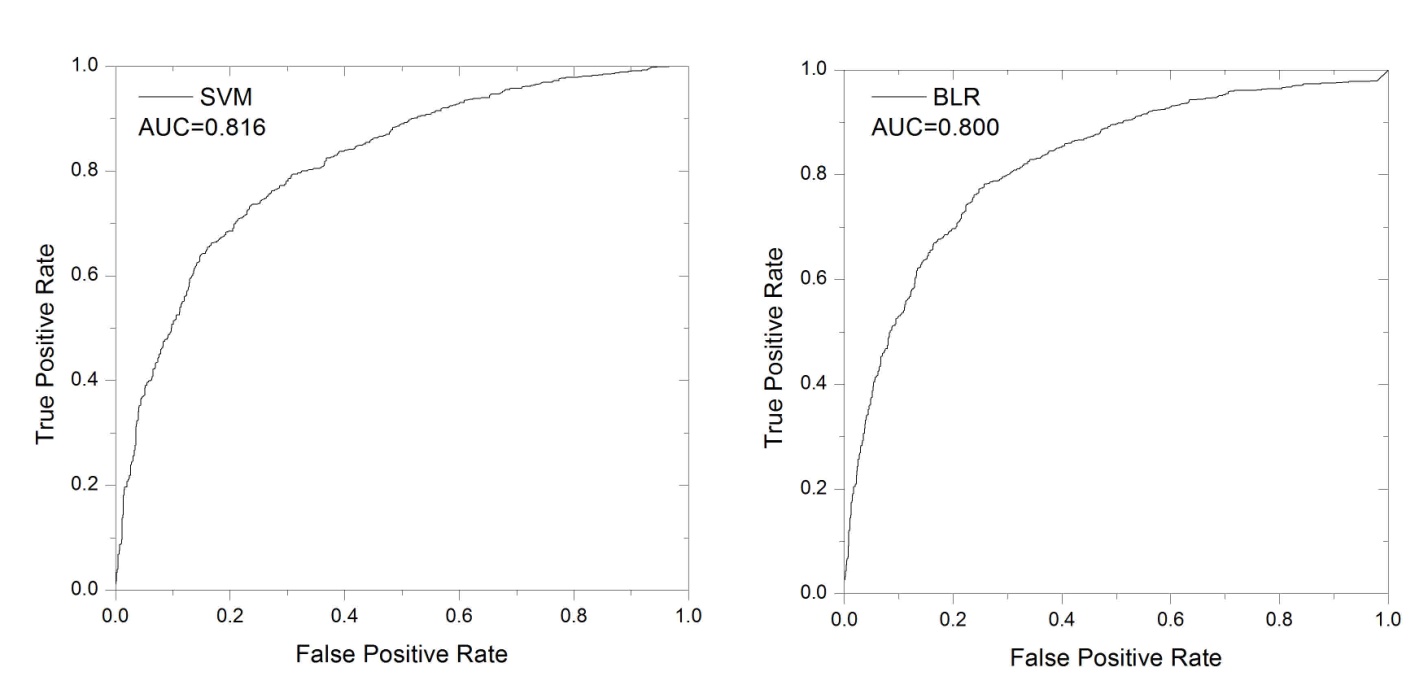
**

Supplement: Figure S1 — The performances of machine learning methods in the identification of HOMA-IR corrected factors in the Cameron Cohort Hispanic Cohort (CCHC). (a) The SVM model; (b) The BLR model. As shown by the area under the receiver operator characteristic curve (AUROC) scores, both methods have good performance in modeling the HOMA-IR corrected factors, while the SVM model (AUC = 0.816) has slightly better performance than the BLR model (AUC = 0.800). (DOCX) [file pone.0021041.s001.docx]
