# Supplementary material for: The Definition of Insulin Resistance Using HOMA-IR for Americans of Mexican Descent Using Machine Learning
Source: PLoS One. 2011 Jun 14;6(6):e21041. doi: 10.1371/journal.pone.0021041 (PMC3114864; doi:10.1371/journal.pone.0021041)
Supplement: Table S1 — Identification of HOMA-IR corrected factors by SVM and BLR. (DOCX) [file pone.0021041.s002.docx]

**Table S1 Identification of HOMA-IR corrected factors by SVM and BLR**

| **Attribute** | **SVM F score ^a^** | **BLR beta value ^b^** |
| --- | --- | --- |
| BMI | 0.189 | 8.833 |
| FPG (mg/dL) | 0.121 | 8.191 |
| waist/hip ratio | 0.065 | 0.857 |
| Serum triglycerides | 0.042 | 3.774 |
| Systolic blood pressure | 0.035 | 1.305 |
| Diastolic blood pressure | 0.035 | 1.910 |
| ALT | 0.028 | 2.594 |
| AST | 0.009 | 0.757 |
| Total cholesterol | 0.001 | 1.436 |
| Gender | 0.000 | ±0.348 |
| High-density lipoprotein (HDL) | 0.030 | -2.858 |
| Low-density lipoprotein (LDL) | 0.006 | -1.236 |
| Heavy physical activity | 0.005 | -1.946 |
| Education level | 0.004 | -0.271 |
| Age | 0.003 | -0.544 |
| alcohol consumption | 0.000 | -0.155 |
| Slight physical activity | 0.000 | -1.484 |
| Annual income | 0.000 | -0.133 |
| Hepatitis history | 0.000 | -1.696 |
| Smoking | 0.000 | -0.030 |
| Moderate physical activity | 0.000 | -2.206 |

^a^ F-score measures the discrimination of two groups, while larger F-score suggests the feature is more discriminative by the variable;

^b^ BLR beta value is the maximum likelihood values of the slope parameters in the BLL model, while larger absolute value suggests larger effect by the factor, and “+/-“ represents the direction of the correlation.
