# Supplementary material for: The Definition of Insulin Resistance Using HOMA-IR for Americans of Mexican Descent Using Machine Learning
Source: PLoS One. 2011 Jun 14;6(6):e21041. doi: 10.1371/journal.pone.0021041 (PMC3114864; doi:10.1371/journal.pone.0021041)
Supplement: Table S2 — Reference ranges of the HOMA-IR corrected factors. (DOCX) [file pone.0021041.s003.docx]

**Table S2 Reference ranges of the HOMA-IR corrected factors**

| **Attribute** | **Reference values** ^b^ |
| --- | --- |
| BMI^a^ | 1: Normal<25; 2: Overweight 25.0-29.9; 3: Obese 30-39.9; 4: Morbid obese ≥40 |
| FPG (mg/dL)^b^ | 1: Normal<100; 2: Pre-diabetic 100-125; 3: Diabetic >125 |
| waist/hip ratio | Males 1: Normal<0.9; 2: High≥0.9  Females 1: Normal<0.8; 2: High≥0.8 |
| Serum triglycerides (mg/dL)^a^ | 1: Normal<150; 2: Borderline high=150-199; 3: High=200–499; 4: Very high≥500 |
| HDL cholesterol (mg/dL) ^a^ | Males 1: Normal≥40; 2: Reduced<40  Females 1: Normal≥50; 2: Reduced<50 |
| Diastolic blood pressure (mmHg)^a^ | 1: Normal<80; 2: Prehypertension=80-89; 3: Hypertension Stage 1=90–99; 4: Hypertension Stage 2≥100 |
| Systolic blood pressure (mmHg)^a^ | 1: Normal<120; 2: Prehypertension=120-139; 3: Hypertension Stage 1=140–159; 4: Hypertension Stage 2≥160 |
| ALT (U/L) | 1: Normal≤40; 2: High>40 |
| AST (U/L) | 1: Normal≤40; 2: High>40 |
| Total cholesterol(mg/dL)^a^ | 1: Normal<200; 2: Borderline high=200-239; 3: High≥240 |

^a^ Reference ranges were based on the recommendations of the American Heart Association ([www.americanheart.org](http://www.americanheart.org)); ^b^ A number was assigned to each category of each factor for the K-means analysis;

^b^ Reference ranges were based on the 2010 Clinical Practice Recommendations of the American Diabetes Association (ADA).
