# Supplementary material for: The Definition of Insulin Resistance Using HOMA-IR for Americans of Mexican Descent Using Machine Learning
Source: PLoS One. 2011 Jun 14;6(6):e21041. doi: 10.1371/journal.pone.0021041 (PMC3114864; doi:10.1371/journal.pone.0021041)
Supplement: Table S3 — The specificity and sensitivity for insulin resistance syndrome of fine-scans of the series of HOMA-IR cutoff values. (DOCX) [file pone.0021041.s004.docx]

**Table S3 The specificity and sensitivity for insulin resistance syndrome of fine-scans of the series of HOMA-IR cutoff values**

| cut_off | Sensitivity | Specificity | MCC |
| --- | --- | --- | --- |
| 0.20 | 1.000 | 0.002 | 0.028 |
| 0.25 | 1.000 | 0.002 | 0.028 |
| 0.30 | 1.000 | 0.004 | 0.040 |
| 0.35 | 1.000 | 0.007 | 0.053 |
| 0.40 | 1.000 | 0.007 | 0.053 |
| 0.45 | 0.999 | 0.011 | 0.060 |
| 0.50 | 0.999 | 0.015 | 0.072 |
| 0.55 | 0.999 | 0.021 | 0.087 |
| 0.60 | 0.999 | 0.025 | 0.098 |
| 0.65 | 0.999 | 0.034 | 0.116 |
| 0.70 | 0.999 | 0.041 | 0.128 |
| 0.75 | 0.999 | 0.048 | 0.141 |
| 0.80 | 0.996 | 0.054 | 0.140 |
| 0.85 | 0.992 | 0.064 | 0.143 |
| 0.90 | 0.992 | 0.075 | 0.159 |
| 0.95 | 0.991 | 0.083 | 0.167 |
| 1.00 | 0.991 | 0.098 | 0.186 |
| 1.05 | 0.991 | 0.112 | 0.204 |
| 1.10 | 0.990 | 0.125 | 0.215 |
| 1.15 | 0.987 | 0.136 | 0.221 |
| 1.20 | 0.986 | 0.150 | 0.234 |
| 1.25 | 0.986 | 0.169 | 0.253 |
| 1.30 | 0.985 | 0.180 | 0.262 |
| 1.35 | 0.980 | 0.194 | 0.265 |
| 1.40 | 0.977 | 0.206 | 0.272 |
| 1.45 | 0.972 | 0.219 | 0.275 |
| 1.50 | 0.965 | 0.244 | 0.286 |
| 1.55 | 0.960 | 0.261 | 0.293 |
| 1.60 | 0.956 | 0.274 | 0.299 |
| 1.65 | 0.951 | 0.292 | 0.308 |
| 1.70 | 0.945 | 0.306 | 0.311 |
| 1.75 | 0.941 | 0.323 | 0.321 |
| 1.80 | 0.932 | 0.336 | 0.320 |
| 1.85 | 0.930 | 0.356 | 0.334 |
| 1.90 | 0.927 | 0.372 | 0.345 |
| 1.95 | 0.919 | 0.382 | 0.344 |
| 2.00 | 0.914 | 0.393 | 0.346 |
| 2.05 | 0.906 | 0.408 | 0.348 |
| 2.10 | 0.899 | 0.423 | 0.354 |
| 2.15 | 0.891 | 0.432 | 0.351 |
| 2.20 | 0.887 | 0.448 | 0.360 |
| 2.25 | 0.877 | 0.464 | 0.362 |
| 2.30 | 0.872 | 0.480 | 0.370 |
| 2.35 | 0.862 | 0.500 | 0.377 |
| 2.40 | 0.853 | 0.512 | 0.377 |
| 2.45 | 0.844 | 0.524 | 0.378 |
| 2.50 | 0.835 | 0.532 | 0.375 |
| 2.55 | 0.825 | 0.544 | 0.375 |
| 2.60 | 0.814 | 0.552 | 0.371 |
| 2.65 | 0.803 | 0.564 | 0.369 |
| 2.70 | 0.794 | 0.574 | 0.369 |
| 2.75 | 0.786 | 0.585 | 0.371 |
| 2.80 | 0.775 | 0.600 | 0.373 |
| 2.85 | 0.766 | 0.613 | 0.377 |
| 2.90 | 0.761 | 0.619 | 0.378 |
| 2.95 | 0.747 | 0.629 | 0.373 |
| 3.00 | 0.742 | 0.636 | 0.374 |
| 3.05 | 0.737 | 0.641 | 0.375 |
| 3.10 | 0.731 | 0.656 | 0.383 |
| 3.15 | 0.717 | 0.669 | 0.382 |
| 3.20 | 0.704 | 0.676 | 0.377 |
| 3.25 | 0.697 | 0.687 | 0.381 |
| 3.30 | 0.688 | 0.700 | 0.385 |
| 3.35 | 0.678 | 0.706 | 0.382 |
| 3.40 | 0.673 | 0.718 | 0.389 |
| 3.45 | 0.664 | 0.725 | 0.388 |
| 3.50 | 0.657 | 0.732 | 0.388 |
| 3.55 | 0.653 | 0.740 | 0.393 |
| 3.60 | 0.642 | 0.749 | 0.392 |
| 3.65 | 0.634 | 0.754 | 0.391 |
| 3.70 | 0.629 | 0.760 | 0.392 |
| 3.75 | 0.624 | 0.769 | 0.397 |
| 3.80 | 0.616 | 0.778 | 0.400 |
| 3.85 | 0.604 | 0.781 | 0.391 |
| 3.90 | 0.595 | 0.785 | 0.387 |
| 3.95 | 0.585 | 0.788 | 0.381 |
| 4.00 | 0.579 | 0.795 | 0.384 |
| 4.05 | 0.560 | 0.800 | 0.372 |
| 4.10 | 0.550 | 0.804 | 0.367 |
| 4.15 | 0.543 | 0.805 | 0.363 |
| 4.20 | 0.530 | 0.809 | 0.355 |
| 4.25 | 0.522 | 0.812 | 0.351 |
| 4.30 | 0.517 | 0.813 | 0.348 |
| 4.35 | 0.513 | 0.819 | 0.351 |
| 4.40 | 0.509 | 0.822 | 0.352 |
| 4.45 | 0.504 | 0.827 | 0.353 |
| 4.50 | 0.498 | 0.832 | 0.353 |
| 4.55 | 0.494 | 0.836 | 0.354 |
| 4.60 | 0.483 | 0.837 | 0.345 |
| 4.65 | 0.478 | 0.840 | 0.345 |
| 4.70 | 0.475 | 0.840 | 0.343 |
| 4.75 | 0.468 | 0.844 | 0.341 |
| 4.80 | 0.463 | 0.849 | 0.342 |
| 4.85 | 0.455 | 0.852 | 0.339 |
| 4.90 | 0.448 | 0.855 | 0.335 |
| 4.95 | 0.442 | 0.855 | 0.329 |
| 5.00 | 0.436 | 0.858 | 0.329 |
| 5.05 | 0.428 | 0.863 | 0.327 |
| 5.10 | 0.421 | 0.866 | 0.325 |
| 5.15 | 0.420 | 0.868 | 0.327 |
| 5.20 | 0.411 | 0.870 | 0.321 |
| 5.25 | 0.406 | 0.874 | 0.323 |
| 5.30 | 0.395 | 0.878 | 0.317 |
| 5.35 | 0.386 | 0.880 | 0.311 |
| 5.40 | 0.384 | 0.883 | 0.313 |
| 5.45 | 0.376 | 0.886 | 0.310 |
| 5.50 | 0.374 | 0.887 | 0.309 |
| 5.55 | 0.366 | 0.889 | 0.304 |
| 5.60 | 0.365 | 0.890 | 0.305 |
| 5.65 | 0.362 | 0.891 | 0.304 |
| 5.70 | 0.360 | 0.894 | 0.306 |
| 5.75 | 0.355 | 0.896 | 0.304 |
| 5.80 | 0.351 | 0.898 | 0.303 |
| 5.85 | 0.350 | 0.899 | 0.303 |
| 5.90 | 0.342 | 0.902 | 0.300 |
| 5.95 | 0.338 | 0.903 | 0.298 |
| 6.00 | 0.335 | 0.904 | 0.296 |
| 6.05 | 0.333 | 0.907 | 0.299 |
| 6.10 | 0.330 | 0.908 | 0.298 |
| 6.15 | 0.330 | 0.909 | 0.300 |
| 6.20 | 0.325 | 0.910 | 0.296 |
| 6.25 | 0.322 | 0.912 | 0.297 |
| 6.30 | 0.318 | 0.913 | 0.294 |
| 6.35 | 0.317 | 0.915 | 0.296 |
| 6.40 | 0.313 | 0.916 | 0.294 |
| 6.45 | 0.311 | 0.918 | 0.294 |
| 6.50 | 0.299 | 0.920 | 0.286 |
| 6.55 | 0.294 | 0.920 | 0.281 |
| 6.60 | 0.292 | 0.921 | 0.280 |
| 6.65 | 0.289 | 0.923 | 0.280 |
| 6.70 | 0.286 | 0.923 | 0.276 |
| 6.75 | 0.284 | 0.925 | 0.280 |
| 6.80 | 0.279 | 0.927 | 0.278 |
| 6.85 | 0.278 | 0.928 | 0.278 |
| 6.90 | 0.273 | 0.928 | 0.273 |
| 6.95 | 0.272 | 0.932 | 0.278 |
| 7.00 | 0.269 | 0.932 | 0.276 |
| 7.05 | 0.263 | 0.935 | 0.274 |
| 7.10 | 0.262 | 0.936 | 0.274 |
| 7.15 | 0.259 | 0.936 | 0.272 |
| 7.20 | 0.258 | 0.936 | 0.270 |
| 7.25 | 0.258 | 0.936 | 0.270 |
| 7.30 | 0.257 | 0.939 | 0.274 |
| 7.35 | 0.253 | 0.940 | 0.272 |
| 7.40 | 0.248 | 0.940 | 0.267 |
| 7.45 | 0.244 | 0.941 | 0.266 |
| 7.50 | 0.243 | 0.943 | 0.268 |
| 7.55 | 0.240 | 0.943 | 0.266 |
| 7.60 | 0.236 | 0.944 | 0.263 |
| 7.65 | 0.234 | 0.944 | 0.260 |
| 7.70 | 0.231 | 0.947 | 0.263 |
| 7.75 | 0.230 | 0.947 | 0.262 |
| 7.80 | 0.225 | 0.947 | 0.256 |
| 7.85 | 0.224 | 0.949 | 0.259 |
| 7.90 | 0.221 | 0.950 | 0.258 |
| 7.95 | 0.219 | 0.951 | 0.257 |
| 8.00 | 0.215 | 0.952 | 0.254 |
| 8.05 | 0.214 | 0.953 | 0.255 |
| 8.10 | 0.214 | 0.953 | 0.255 |
| 8.15 | 0.209 | 0.953 | 0.249 |
| 8.20 | 0.204 | 0.955 | 0.247 |
| 8.25 | 0.199 | 0.956 | 0.244 |
| 8.30 | 0.196 | 0.957 | 0.243 |
| 8.35 | 0.195 | 0.957 | 0.241 |
| 8.40 | 0.192 | 0.957 | 0.238 |
| 8.45 | 0.192 | 0.960 | 0.246 |
| 8.50 | 0.191 | 0.961 | 0.247 |
| 8.55 | 0.191 | 0.961 | 0.247 |
| 8.60 | 0.189 | 0.962 | 0.246 |
| 8.65 | 0.185 | 0.962 | 0.242 |
| 8.70 | 0.185 | 0.962 | 0.242 |
| 8.75 | 0.180 | 0.962 | 0.236 |
| 8.80 | 0.180 | 0.963 | 0.238 |
| 8.85 | 0.177 | 0.963 | 0.235 |
| 8.90 | 0.175 | 0.965 | 0.236 |
| 8.95 | 0.175 | 0.965 | 0.236 |
| 9.00 | 0.175 | 0.965 | 0.236 |
| 9.05 | 0.174 | 0.965 | 0.235 |
| 9.10 | 0.172 | 0.965 | 0.233 |
| 9.15 | 0.169 | 0.965 | 0.229 |
| 9.20 | 0.167 | 0.965 | 0.227 |
| 9.25 | 0.166 | 0.965 | 0.225 |
| 9.30 | 0.166 | 0.965 | 0.225 |
| 9.35 | 0.165 | 0.965 | 0.224 |
| 9.40 | 0.164 | 0.965 | 0.222 |
| 9.45 | 0.161 | 0.965 | 0.219 |
| 9.50 | 0.158 | 0.965 | 0.216 |
| 9.55 | 0.155 | 0.965 | 0.211 |
| 9.60 | 0.151 | 0.965 | 0.206 |
| 9.65 | 0.150 | 0.965 | 0.205 |
| 9.70 | 0.147 | 0.969 | 0.211 |
| 9.75 | 0.147 | 0.971 | 0.215 |
| 9.80 | 0.146 | 0.971 | 0.214 |
| 9.85 | 0.145 | 0.971 | 0.212 |
| 9.90 | 0.145 | 0.971 | 0.212 |
| 9.95 | 0.143 | 0.971 | 0.210 |
| 10.00 | 0.141 | 0.971 | 0.207 |
| 10.05 | 0.141 | 0.971 | 0.207 |
| 10.10 | 0.140 | 0.972 | 0.208 |
| 10.15 | 0.136 | 0.972 | 0.203 |
| 10.20 | 0.135 | 0.972 | 0.201 |
| 10.25 | 0.135 | 0.972 | 0.201 |
| 10.30 | 0.133 | 0.972 | 0.199 |
| 10.35 | 0.132 | 0.972 | 0.198 |
| 10.40 | 0.132 | 0.972 | 0.198 |
| 10.45 | 0.131 | 0.975 | 0.203 |
| 10.50 | 0.130 | 0.975 | 0.204 |
| 10.55 | 0.128 | 0.976 | 0.205 |
| 10.60 | 0.128 | 0.976 | 0.205 |
| 10.65 | 0.127 | 0.976 | 0.203 |
| 10.70 | 0.125 | 0.976 | 0.200 |
| 10.75 | 0.123 | 0.977 | 0.201 |
| 10.80 | 0.122 | 0.977 | 0.199 |
| 10.85 | 0.122 | 0.977 | 0.199 |
| 10.90 | 0.121 | 0.977 | 0.197 |
| 10.95 | 0.121 | 0.977 | 0.197 |
| 11.00 | 0.121 | 0.977 | 0.197 |
| 11.05 | 0.121 | 0.978 | 0.200 |
| 11.10 | 0.121 | 0.979 | 0.203 |
| 11.15 | 0.121 | 0.979 | 0.203 |
| 11.20 | 0.118 | 0.979 | 0.199 |
| 11.25 | 0.118 | 0.979 | 0.199 |
| 11.30 | 0.116 | 0.979 | 0.196 |
| 11.35 | 0.114 | 0.980 | 0.197 |
| 11.40 | 0.113 | 0.980 | 0.195 |
| 11.45 | 0.112 | 0.980 | 0.193 |
| 11.50 | 0.111 | 0.980 | 0.191 |
| 11.55 | 0.109 | 0.980 | 0.189 |
| 11.60 | 0.107 | 0.980 | 0.186 |
| 11.65 | 0.106 | 0.981 | 0.187 |
| 11.70 | 0.102 | 0.981 | 0.181 |
| 11.75 | 0.099 | 0.981 | 0.177 |
| 11.80 | 0.099 | 0.982 | 0.180 |
| 11.85 | 0.098 | 0.982 | 0.178 |
| 11.90 | 0.098 | 0.983 | 0.181 |
| 11.95 | 0.097 | 0.984 | 0.182 |
| 12.00 | 0.096 | 0.984 | 0.180 |
| 12.05 | 0.094 | 0.984 | 0.178 |
| 12.10 | 0.093 | 0.984 | 0.176 |
| 12.15 | 0.093 | 0.985 | 0.180 |
| 12.20 | 0.093 | 0.985 | 0.180 |
| 12.25 | 0.091 | 0.985 | 0.176 |
| 12.30 | 0.091 | 0.985 | 0.176 |
| 12.35 | 0.088 | 0.985 | 0.172 |
| 12.40 | 0.084 | 0.985 | 0.166 |
| 12.45 | 0.082 | 0.985 | 0.161 |
| 12.50 | 0.077 | 0.985 | 0.153 |
| 12.55 | 0.077 | 0.987 | 0.160 |
| 12.60 | 0.075 | 0.987 | 0.157 |
| 12.65 | 0.075 | 0.987 | 0.157 |
| 12.70 | 0.074 | 0.987 | 0.155 |
| 12.75 | 0.074 | 0.988 | 0.159 |
| 12.80 | 0.073 | 0.988 | 0.156 |
| 12.85 | 0.073 | 0.988 | 0.156 |
| 12.90 | 0.072 | 0.988 | 0.154 |
| 12.95 | 0.070 | 0.988 | 0.152 |
| 13.00 | 0.069 | 0.988 | 0.150 |
| 13.05 | 0.067 | 0.988 | 0.145 |
| 13.10 | 0.064 | 0.988 | 0.141 |
| 13.15 | 0.063 | 0.988 | 0.138 |
| 13.20 | 0.063 | 0.988 | 0.138 |
| 13.25 | 0.063 | 0.988 | 0.138 |
| 13.30 | 0.062 | 0.988 | 0.136 |
| 13.35 | 0.062 | 0.988 | 0.136 |
| 13.40 | 0.062 | 0.988 | 0.136 |
| 13.45 | 0.062 | 0.989 | 0.140 |
| 13.50 | 0.060 | 0.989 | 0.137 |
| 13.55 | 0.059 | 0.989 | 0.135 |
| 13.60 | 0.059 | 0.989 | 0.135 |
| 13.65 | 0.058 | 0.989 | 0.132 |
| 13.70 | 0.057 | 0.989 | 0.130 |
| 13.75 | 0.057 | 0.989 | 0.130 |
| 13.80 | 0.057 | 0.989 | 0.130 |
| 13.85 | 0.054 | 0.989 | 0.125 |
| 13.90 | 0.054 | 0.989 | 0.125 |
| 13.95 | 0.054 | 0.990 | 0.129 |
| 14.00 | 0.053 | 0.990 | 0.126 |
| 14.05 | 0.052 | 0.990 | 0.123 |
| 14.10 | 0.052 | 0.991 | 0.127 |
| 14.15 | 0.050 | 0.991 | 0.125 |
| 14.20 | 0.050 | 0.991 | 0.125 |
| 14.25 | 0.050 | 0.991 | 0.125 |
| 14.30 | 0.050 | 0.991 | 0.125 |
| 14.35 | 0.050 | 0.991 | 0.125 |
| 14.40 | 0.050 | 0.991 | 0.125 |
| 14.45 | 0.049 | 0.991 | 0.122 |
| 14.50 | 0.049 | 0.991 | 0.122 |
| 14.55 | 0.048 | 0.991 | 0.120 |
| 14.60 | 0.048 | 0.991 | 0.120 |
| 14.65 | 0.048 | 0.991 | 0.120 |
| 14.70 | 0.048 | 0.991 | 0.120 |
| 14.75 | 0.048 | 0.992 | 0.124 |
| 14.80 | 0.048 | 0.992 | 0.124 |
| 14.85 | 0.048 | 0.992 | 0.124 |
| 14.90 | 0.048 | 0.992 | 0.124 |
| 14.95 | 0.048 | 0.992 | 0.124 |
| 15.00 | 0.047 | 0.992 | 0.121 |
| 15.05 | 0.047 | 0.992 | 0.121 |
| 15.10 | 0.047 | 0.992 | 0.121 |
| 15.15 | 0.047 | 0.992 | 0.121 |
| 15.20 | 0.045 | 0.992 | 0.118 |
| 15.25 | 0.045 | 0.992 | 0.118 |
| 15.30 | 0.045 | 0.992 | 0.118 |
| 15.35 | 0.045 | 0.992 | 0.118 |
| 15.40 | 0.045 | 0.992 | 0.118 |
| 15.45 | 0.045 | 0.992 | 0.118 |
| 15.50 | 0.045 | 0.992 | 0.118 |
| 15.55 | 0.044 | 0.992 | 0.116 |
| 15.60 | 0.044 | 0.992 | 0.116 |
| 15.65 | 0.044 | 0.992 | 0.116 |
| 15.70 | 0.044 | 0.992 | 0.116 |
| 15.75 | 0.044 | 0.992 | 0.116 |
| 15.80 | 0.044 | 0.992 | 0.116 |
| 15.85 | 0.044 | 0.992 | 0.116 |
| 15.90 | 0.043 | 0.992 | 0.113 |
| 15.95 | 0.043 | 0.992 | 0.113 |
| 16.00 | 0.042 | 0.992 | 0.110 |
| 16.05 | 0.040 | 0.992 | 0.107 |
| 16.10 | 0.040 | 0.992 | 0.111 |
| 16.15 | 0.040 | 0.992 | 0.111 |
| 16.20 | 0.040 | 0.992 | 0.111 |
| 16.25 | 0.040 | 0.993 | 0.116 |
| 16.30 | 0.040 | 0.993 | 0.116 |
| 16.35 | 0.040 | 0.993 | 0.116 |
| 16.40 | 0.040 | 0.993 | 0.116 |
| 16.45 | 0.039 | 0.993 | 0.113 |
| 16.50 | 0.039 | 0.993 | 0.113 |
| 16.55 | 0.039 | 0.994 | 0.118 |
| 16.60 | 0.039 | 0.994 | 0.118 |
| 16.65 | 0.039 | 0.994 | 0.118 |
| 16.70 | 0.039 | 0.994 | 0.118 |
| 16.75 | 0.039 | 0.994 | 0.118 |
| 16.80 | 0.039 | 0.994 | 0.118 |
| 16.85 | 0.039 | 0.994 | 0.118 |
| 16.90 | 0.039 | 0.994 | 0.118 |
| 16.95 | 0.039 | 0.994 | 0.118 |
| 17.00 | 0.039 | 0.994 | 0.118 |
| 17.05 | 0.038 | 0.994 | 0.115 |
| 17.10 | 0.038 | 0.994 | 0.115 |
| 17.15 | 0.038 | 0.994 | 0.115 |
| 17.20 | 0.038 | 0.994 | 0.115 |
| 17.25 | 0.038 | 0.994 | 0.115 |
| 17.30 | 0.036 | 0.994 | 0.112 |
| 17.35 | 0.036 | 0.994 | 0.112 |
| 17.40 | 0.036 | 0.994 | 0.112 |
| 17.45 | 0.036 | 0.994 | 0.112 |
| 17.50 | 0.036 | 0.994 | 0.112 |
| 17.55 | 0.036 | 0.994 | 0.112 |
| 17.60 | 0.034 | 0.994 | 0.106 |
| 17.65 | 0.034 | 0.995 | 0.111 |
| 17.70 | 0.034 | 0.995 | 0.111 |
| 17.75 | 0.031 | 0.995 | 0.105 |
| 17.80 | 0.031 | 0.995 | 0.105 |
| 17.85 | 0.030 | 0.995 | 0.102 |
| 17.90 | 0.030 | 0.995 | 0.102 |
| 17.95 | 0.030 | 0.995 | 0.102 |
| 18.00 | 0.029 | 0.995 | 0.098 |
| 18.05 | 0.029 | 0.995 | 0.098 |
| 18.10 | 0.029 | 0.995 | 0.098 |
| 18.15 | 0.029 | 0.995 | 0.098 |
| 18.20 | 0.028 | 0.995 | 0.095 |
| 18.25 | 0.028 | 0.995 | 0.095 |
| 18.30 | 0.028 | 0.996 | 0.101 |
| 18.35 | 0.028 | 0.996 | 0.101 |
| 18.40 | 0.028 | 0.996 | 0.101 |
| 18.45 | 0.028 | 0.996 | 0.101 |
| 18.50 | 0.028 | 0.996 | 0.101 |
| 18.55 | 0.028 | 0.996 | 0.101 |
| 18.60 | 0.028 | 0.996 | 0.101 |
| 18.65 | 0.028 | 0.996 | 0.101 |
| 18.70 | 0.028 | 0.996 | 0.101 |
| 18.75 | 0.028 | 0.996 | 0.101 |
| 18.80 | 0.028 | 0.996 | 0.101 |
| 18.85 | 0.026 | 0.996 | 0.097 |
| 18.90 | 0.026 | 0.997 | 0.103 |
| 18.95 | 0.026 | 0.997 | 0.103 |
| 19.00 | 0.026 | 0.997 | 0.103 |
| 19.05 | 0.026 | 0.997 | 0.103 |
| 19.10 | 0.026 | 0.997 | 0.103 |
| 19.15 | 0.026 | 0.997 | 0.103 |
| 19.20 | 0.026 | 0.997 | 0.103 |
| 19.25 | 0.026 | 0.997 | 0.103 |
| 19.30 | 0.026 | 0.997 | 0.103 |
| 19.35 | 0.026 | 0.997 | 0.103 |
| 19.40 | 0.026 | 0.997 | 0.103 |
| 19.45 | 0.026 | 0.997 | 0.103 |
| 19.50 | 0.026 | 0.997 | 0.103 |
| 19.55 | 0.026 | 0.997 | 0.103 |
| 19.60 | 0.025 | 0.997 | 0.100 |
| 19.65 | 0.025 | 0.997 | 0.100 |
| 19.70 | 0.024 | 0.997 | 0.096 |
| 19.75 | 0.024 | 0.998 | 0.103 |
| 19.80 | 0.024 | 0.998 | 0.103 |
| 19.85 | 0.024 | 0.998 | 0.103 |
| 19.90 | 0.023 | 0.998 | 0.099 |
| 19.95 | 0.023 | 0.998 | 0.099 |
| 20.00 | 0.021 | 0.998 | 0.096 |
| 20.05 | 0.020 | 0.998 | 0.092 |
| 20.10 | 0.020 | 0.998 | 0.092 |
| 20.15 | 0.020 | 0.998 | 0.092 |
| 20.20 | 0.019 | 0.998 | 0.088 |
| 20.25 | 0.019 | 0.998 | 0.088 |
| 20.30 | 0.019 | 0.998 | 0.088 |
| 20.35 | 0.019 | 0.998 | 0.088 |
| 20.40 | 0.019 | 0.998 | 0.088 |
| 20.45 | 0.019 | 0.998 | 0.088 |
| 20.50 | 0.019 | 0.998 | 0.088 |
| 20.55 | 0.019 | 0.998 | 0.088 |
| 20.60 | 0.019 | 0.998 | 0.088 |
| 20.65 | 0.019 | 0.998 | 0.088 |
| 20.70 | 0.019 | 0.998 | 0.088 |
| 20.75 | 0.019 | 0.998 | 0.088 |
| 20.80 | 0.019 | 0.998 | 0.088 |
| 20.85 | 0.019 | 0.998 | 0.088 |
| 20.90 | 0.019 | 0.998 | 0.088 |
| 20.95 | 0.019 | 0.998 | 0.088 |
| 21.00 | 0.019 | 0.998 | 0.088 |
| 21.05 | 0.019 | 0.998 | 0.088 |
| 21.10 | 0.019 | 0.998 | 0.088 |
| 21.15 | 0.019 | 0.998 | 0.088 |
| 21.20 | 0.019 | 0.998 | 0.088 |
| 21.25 | 0.018 | 0.998 | 0.084 |
| 21.30 | 0.018 | 0.998 | 0.084 |
| 21.35 | 0.018 | 0.998 | 0.084 |
| 21.40 | 0.018 | 0.998 | 0.084 |
| 21.45 | 0.018 | 0.998 | 0.084 |
| 21.50 | 0.018 | 0.998 | 0.084 |
| 21.55 | 0.018 | 0.998 | 0.084 |
| 21.60 | 0.018 | 0.998 | 0.084 |
| 21.65 | 0.018 | 0.998 | 0.084 |
| 21.70 | 0.016 | 0.998 | 0.080 |
| 21.75 | 0.016 | 0.998 | 0.080 |
| 21.80 | 0.016 | 0.998 | 0.080 |
| 21.85 | 0.016 | 0.998 | 0.080 |
| 21.90 | 0.016 | 0.998 | 0.080 |
| 21.95 | 0.015 | 0.998 | 0.075 |
| 22.00 | 0.015 | 0.998 | 0.075 |
| 22.05 | 0.015 | 0.998 | 0.075 |
| 22.10 | 0.015 | 0.998 | 0.075 |
| 22.15 | 0.015 | 0.998 | 0.075 |
| 22.20 | 0.015 | 0.998 | 0.075 |
| 22.25 | 0.015 | 0.998 | 0.075 |
| 22.30 | 0.015 | 0.998 | 0.075 |
| 22.35 | 0.015 | 0.998 | 0.075 |
| 22.40 | 0.015 | 0.998 | 0.075 |
| 22.45 | 0.015 | 0.998 | 0.075 |
| 22.50 | 0.015 | 0.998 | 0.075 |
| 22.55 | 0.014 | 0.998 | 0.071 |
| 22.60 | 0.014 | 0.998 | 0.071 |
| 22.65 | 0.014 | 0.998 | 0.071 |
| 22.70 | 0.013 | 0.998 | 0.066 |
| 22.75 | 0.013 | 0.998 | 0.066 |
| 22.80 | 0.013 | 0.998 | 0.066 |
| 22.85 | 0.013 | 0.998 | 0.066 |
| 22.90 | 0.013 | 0.998 | 0.066 |
| 22.95 | 0.013 | 0.998 | 0.066 |
| 23.00 | 0.013 | 0.998 | 0.066 |
| 23.05 | 0.013 | 0.998 | 0.066 |
| 23.10 | 0.013 | 0.998 | 0.066 |
| 23.15 | 0.013 | 0.998 | 0.066 |
| 23.20 | 0.013 | 0.999 | 0.075 |
| 23.25 | 0.013 | 0.999 | 0.075 |
| 23.30 | 0.013 | 0.999 | 0.075 |
| 23.35 | 0.011 | 0.999 | 0.070 |
| 23.40 | 0.011 | 0.999 | 0.070 |
| 23.45 | 0.011 | 0.999 | 0.070 |
| 23.50 | 0.011 | 0.999 | 0.070 |
| 23.55 | 0.011 | 0.999 | 0.070 |
| 23.60 | 0.011 | 0.999 | 0.070 |
| 23.65 | 0.011 | 0.999 | 0.070 |
| 23.70 | 0.011 | 0.999 | 0.070 |
| 23.75 | 0.010 | 0.999 | 0.065 |
| 23.80 | 0.010 | 0.999 | 0.065 |
| 23.85 | 0.010 | 0.999 | 0.065 |
| 23.90 | 0.010 | 0.999 | 0.065 |
| 23.95 | 0.010 | 0.999 | 0.065 |
| 24.00 | 0.010 | 0.999 | 0.065 |
| 24.05 | 0.010 | 0.999 | 0.065 |
| 24.10 | 0.010 | 0.999 | 0.065 |
| 24.15 | 0.010 | 0.999 | 0.065 |
| 24.20 | 0.010 | 0.999 | 0.065 |
| 24.25 | 0.010 | 0.999 | 0.065 |
| 24.30 | 0.009 | 0.999 | 0.059 |
| 24.35 | 0.009 | 0.999 | 0.059 |
| 24.40 | 0.009 | 0.999 | 0.059 |
| 24.45 | 0.009 | 0.999 | 0.059 |
| 24.50 | 0.009 | 0.999 | 0.059 |
| 24.55 | 0.009 | 0.999 | 0.059 |
| 24.60 | 0.009 | 0.999 | 0.059 |
| 24.65 | 0.008 | 0.999 | 0.053 |
| 24.70 | 0.008 | 0.999 | 0.053 |
| 24.75 | 0.008 | 0.999 | 0.053 |
| 24.80 | 0.008 | 0.999 | 0.053 |
| 24.85 | 0.008 | 0.999 | 0.053 |
| 24.90 | 0.008 | 0.999 | 0.053 |
| 24.95 | 0.008 | 0.999 | 0.053 |
| 25.00 | 0.008 | 0.999 | 0.053 |
